# Supplementary material for: Effectiveness of Resistance Training Program on Body Composition in Adults Following Vegan Diet versus Omnivorous Diet; Developed in Mobile Health Modality
Source: Nutrients. 2024 Aug 2;16(15):2539. doi: 10.3390/nu16152539 (PMC11314574; doi:10.3390/nu16152539)
Supplement: Supplementary file 1 [file nutrients-16-02539-s001.zip › nutrients-3094551-supplementary.pdf]

Supplementary Table S1: Ancova BMI Analysis

Post intervention BMI adjusted by Baseline BMI	Estimate	Std. Error	t value	Lower	Uper	P
VEG_D-C v/s VEG_D- RT_P	0.756	0.303	2.500	-0.042	1.554	0.070
OMN_D- RT_P v/s VEG_D- RT_P	0.548	0.327	1.673	-0.315	1.411	0.346
OMN_D-C v/s VEG_D- RT_P	1.046	0.327	3.204	0.186	1.907	0.011*
OMN_D- RT_P v/s VEG_D-C	-0.208	0.316	-0.658	-1.042	0.626	0.912
OMN_D-C v/s VEG_D-C	0.291	0.303	0.958	-0.509	1.090	0.773
OMN_D-C v/s OMN_D- RT_P	0.500	0.337	1.481	-0.389	1.387	0.454
**BMI**= Boda Mass Index; **VEG_D-RT_P**= Vegan Diet Resistance Training Program; **VEG_D-C**= Vegan Diet Control; **OMN_D-RT_P**= Omnivore Diet Resistance Training Program; **OMN_D-C**= Omnivore Control;						

Supplementary Table S2: Ancova KFM Analysis

Post intervention KFM adjusted by Baseline KFM	Estimate	Std. Error	t value	Lower	Uper	P
VEG_D-C v/s VEG_D- RT_P	2.078	0.702	2.961	0.228	3.928	0.022*
OMN_D- RT_P v/s VEG_D- RT_P	1.055	0.758	1.392	-0.944	3.055	0.508
OMN_D-C v/s VEG_D- RT_P	3.192	0.756	4.220	1.198	5.186	<0.001*
OMN_D- RT_P v/s VEG_D-C	-1.023	0.731	-1.399	-2.950	0.905	0.504
OMN_D-C v/s VEG_D-C	1.114	0.701	1.589	-0.734	2.962	0.391
OMN_D-C v/s OMN_D- RT_P	2.137	0.778	2.746	0.085	4.188	0.038 *
**KFM**= Kilograms Fat Mass; **VEG_D-RT_P**= Vegan Diet Resistance Training Program; **VEG_D-C**= Vegan Diet Control; **OMN_D-RT_P**= Omnivore Diet Resistance Training Program; **OMN_D-C**= Omnivore Control.						
